# Primary resistance to cetuximab therapy in *EGFR* FISH-positive colorectal cancer patients

**DOI:** 10.1038/sj.bjc.6604439

**Published:** 2008-06-24

**Authors:** F Cappuzzo, M Varella-Garcia, G Finocchiaro, M Skokan, S Gajapathy, C Carnaghi, L Rimassa, E Rossi, C Ligorio, L Di Tommaso, A J Holmes, L Toschi, G Tallini, A Destro, M Roncalli, A Santoro, P A Jänne

**Affiliations:** 1 Department of Medical Oncology, Istituto Clinico Humanitas IRCCS, Milan University, Rozzano, Italy; 2 Department of Medicine/Medical Oncology, University of Colorado Cancer Center, Aurora, CO, USA; 3 CINECA-Interuniversity Consortium, Bologna, Italy; 4 Pathology Unit, Bologna University, Bellaria Hospital, Bologna, Italy; 5 Department of Medical Oncology, Dana-Farber Cancer Institute, Boston, MA, USA; 6 Pathology Unit, Istituto Clinico Humanitas IRCCS, Milan University, Rozzano, Italy

**Keywords:** cetuximab, *EGFR*, *KRAS*, *BRAF*, *MET*, *IGF1R*

## Abstract

The impact of *KRAS* mutations on cetuximab sensitivity in epidermal growth factor receptor fluorescence *in situ* hybridisation-positive (*EGFR* FISH+) metastatic colorectal cancer patients (mCRC) has not been previously investigated. In the present study, we analysed *KRAS*, *BRAF*, *PI3KCA*, *MET*, and *IGF1R* in 85 mCRC treated with cetuximab-based therapy in whom *EGFR* status was known. *KRAS* mutations (52.5%) negatively affected response only in *EGFR* FISH+ patients. *EGFR* FISH+/*KRAS* mutated had a significantly lower response rate (*P*=0.04) than *EGFR* FISH+/*KRAS* wild type patients. Four *EGFR* FISH+ patients with *KRAS* mutations responded to cetuximab therapy. *BRAF* was mutated in 5.0% of patients and none responded to the therapy. *PI3KCA* mutations (17.7%) were not associated to cetuximab sensitivity. Patients overexpressing IGF1R (74.3%) had significantly longer survival than patients with low IGF1R expression (*P*=0.006), with no difference in response rate. *IGF1R* gene amplification was not detected, and only two (2.6%) patients, both responders, had *MET* gene amplification. In conclusion, *KRAS* mutations are associated with cetuximab failure in *EGFR* FISH+ mCRC, even if it does not preclude response. The rarity of *MET* and *IGF1R* gene amplification suggests a marginal role in primary resistance. The potential prognostic implication of IGF1R expression merits further evaluation.

With about 400 000 cases each year, colorectal cancer (CRC) is one of the most common human malignancies and one of the leading causes of cancer-related death in the western world (Jemal *et al*, 2007). During the last decade, median duration of survival among patients with metastatic CRC (mCRC) has increased from 12 to 21 months, mainly because of the introduction of new cytotoxic agents such as oxaliplatin and irinotecan (De Gramont *et al*, 2000; Douillard *et al*, 2000).

Improvements in the knowledge of cancer biology has led to the development of agents that specifically inhibit tumour growth. The epidermal growth factor receptor (*EGFR*) is often upregulated in CRC, and monoclonal antibodies represent one of the most important options to inhibit such a target. Cetuximab (Erbitux, Merck, Lyon, France), a monoclonal antibody interfering with the extracellular domain of *EGFR*, has proven to be active in *EGFR* expressing CRC in whom other treatments have failed (Jonker *et al*, 2007). However, only a small proportion of patients (<20%) achieve an objective response, and tumour shrinkage is not confined to patients with high level of EGFR protein expression determined by immunohistochemistry (IHC, Chung *et al*, 2005).

Recently, several studies provided relevant insights on mechanisms underlying cetuximab sensitivity. Retrospective analyses showed that tumour regression is more frequently observed among patients with increased *EGFR* gene copy number (Moroni *et al*, 2005; Sartore-Bianchi *et al*, 2007; Cappuzzo *et al*, 2008), while lack of response and short survival was observed in individuals harbouring a *KRAS* mutation (Lièvre *et al*, 2006, 2008; Di Fiore *et al*, 2007; Khambata-Ford *et al*, 2007; Amado *et al*, 2008; De Roock *et al*, 2008). Nevertheless, the incidence of *KRAS* mutations in CRC cannot account for all resistant cases (Bamford *et al*, 2004), and a consistent proportion of patients with increased *EGFR* gene copy number does not respond to the therapy (Sartore-Bianchi *et al*, 2007; Cappuzzo *et al*, 2008). For instance, in our previous experience (Cappuzzo *et al*, 2008), disease progression was observed in 39.5% of patients with *EGFR* gene gain detected by fluorescence *in situ* hybridisation (*EGFR* FISH+).

Several preclinical findings suggest that *MET*, the hepatocyte growth factor receptor, could interfere with anti-*EGFR* strategies. *MET* is a tyrosine kinase receptor (RTK) involved in cellular proliferation and apoptosis (Jiang *et al*, 1999). Activation of *MET* may lead to the activation of pathways downstream of *Ras* (Graziani *et al*, 1991; Halaban *et al*, 1992; Rodriguez-Viciana *et al*, 1994), such as the Raf/MEK/mitogen-activated protein kinase (MAPK) and the phosphatidylinositol 3-kinase (PI3K)/protein kinase B pathway (PKB). In addition, *MET* is able to directly activate PI3K/PKB pathway in a *Ras* independent manner (Ponzetto *et al*, 1993). Concomitant RTK upregulation including *MET* is common in human carcinomas with high frequency of *KRAS* mutations, including colorectal cancer (Long *et al*, 2003). Moreover, studies in lung cancer have shown that *MET* gene amplification is responsible for acquired resistance to EGFR-tyrosine kinase inhibitors (Engelman *et al*, 2007).

The insulin-like growth factor receptor 1 (IGF1R) is a tetrameric transmembrane RTK implicated in promoting oncogenic transformation, growth, and survival of cancer cells (Baserga *et al*, 1997; Blakesley *et al*, 1997; Dufourny *et al*, 1997; Khandwala *et al*, 2000). IGF1R activation triggers a cascade of reactions involving the Raf/MEK/MAPK and the PI3K/PKB pathways (LeRoith *et al*, 1995; Jones and Clemmons, 1995). Data on glioblastoma cell lines suggested that *IGF1R* mediates resistance to anti-EGFR therapy through continued activation of the PI3K-*AKT* pathway (Chakravarti *et al*, 2002). *IGF1R* results in upregulation in the majority of CRC, most likely contributing to the aggressive growth characteristics of these tumours and poor prognosis (Hakam *et al*, 1999; Weber *et al*, 2002).

The impact of *KRAS* mutations on cetuximab sensitivity in *EGFR* FISH+ patients has not been previously investigated. Moreover, no clinical data exist on whether *MET* or *IGF1R* gene gain could interfere with cetuximab sensitivity. On the basis of these premises, we decided to conduct a study exploring the impact of different biomarkers, including *KRAS*, *MET* and *IGF1R*, on primary resistance to cetuximab therapy in metastatic, chemorefractory, and CRC with known *EGFR* FISH status.

## Materials and methods

### Patient selection

The present study was conducted in a cohort of 85 chemorefractory, mCRC patients exposed to cetuximab-based therapy and previously evaluated for *EGFR* by FISH (Cappuzzo *et al*, 2008). Briefly, in our previous experience, patients were selected based on two main criteria: presence of at least one measurable lesion and availability of tumour tissue. No other clinical or biological criterion was used for patient selection. Cetuximab was given to each patient at the initial dose of 400 mg per square meter, followed by weekly infusion of 250 mg per square meter. In all patients, disease assessment was performed every 2 months, with a confirmatory evaluation no less than 4 weeks after the response assessment, according to the RECIST criteria (Therasse *et al*, 2000). The whole study population included 85 patients all pretreated with chemotherapy, including irinotecan (83.5%) and/or oxaliplatin (84.7%). The majority of patients were male (63.5%) with a performance status of 0–1 (96.5%), and with a median age of 63.2 years, with a primitive tumour in colon (76.5%) or rectum (23.5%). *EGFR* was assessed by FISH in tumour samples from primary tumour (43 cases), from metastasis (20 cases) or both primary tumour and corresponding metastasis (22 cases). A mean ⩾2.92 *EGFR* gene copy number qualified the sample as *EGFR* FISH+. Among the 85 evaluable patients, 43 (50.6%) were *EGFR* FISH+ and 42 (49.4%) were *EGFR* FISH−. Cetuximab therapy produced a significantly higher response rate (RR, 32.5 *vs* 2.3%, *P*<0.0001) and a significantly longer time to progression (TTP, 6.6 *vs* 3.5 months, *P*=0.02) in *EGFR* FISH+ than in *EGFR* FISH− patients. The study was approved by the local Ethics Committee and was conducted in accordance with ethical principles stated in the most recent version of the Declaration of Helsinki.

### Tissue preparation and IHC analysis

Sections from paraffin-embedded tissue blocks containing representative malignant cells obtained at time of diagnosis were used for this analysis. Sections were stained with antibodies against IGF1R (Novus Biologicals, Littleton, CO, USA) according to the manufacturer's recommended protocols. Briefly, 4 μm-thick tissue sections were placed on glass slides and deparaffinised. The tissue sections were incubated in 1 mM EDTA (pH 8) for 40 min at 98°C to unmask the antigens. The sections were then incubated with IGF1R mouse antibody (1 : 50 diluted in phosphate buffer).

Immunohistochemical staining was performed at the Pathology Department of Istituto Clinico Humanitas, Italy and slides were interpreted independently by two pathologists (CL and LDT) who were blinded to all patient information. As previously described for IGF1R in lung cancer (Cappuzzo *et al*, 2006), a semiquantitative approach was used to generate a score for each tissue core. The percentage of stained cells (0–100%) was multiplied by the dominant intensity pattern of staining, considering one as negative or trace, two as weak, three as moderate and four as strong. Therefore, the overall score ranged from 0 to 400.

### FISH analyses

Blank tumour sections were submitted to dual-colour FISH assays using in-house developed IGF1R/CEP15 and MET/CEN7 probe cocktails on separate slides for each patient. The assays were performed according to standard laboratory protocol. Initially the slides were incubated for 2 h – overnight at 65°C, deparaffinised in Citro-Solv (Fisher) and washed in 100% ethanol for 5 min. The slides were incubated in 2XSSC at 75°C for 6–22 min and digested in 0.25 mg ml^−1^ Proteinase K/2XSSC at 45°C for 6–25 min. Then, the slides were washed in 2XSSC for 5 min and dehydrated in ethanol. Probes were applied to the selected hybridisation areas (150–300 ng of labelled DNA for each specimen), which were covered with glass coverslips and sealed with rubber cement. DNA denaturation was performed for 15 min at 80°C and the slides were incubated at 37°C for 40–48 h.

Posthybridisation washes were performed with 2XSSC/0.3% NP-40 at 72°C for 2 min. Then, the slides were washed in 2XSSC for 1 min and dehydrated in ethanol. Chromatin was counterstained with DAPI (0.3 *μ*g ml^−1^ in Vectashield, Vector Laboratories). Analysis was performed on epifluorescence microscopes using single interference filter sets for green (FITC), red (Texas red), aqua (Aqua) and blue (DAPI) as well as dual (red/green) and triple (blue, red, green) band pass filters. Each specimen was evaluated in eight different tumour foci with 10 cells analysed in each area (total of 80 cells per specimen).

### Mutation analyses

Additional biomarkers analysed in the present study included *KRAS*, *BRAF*, PI3KCA, and *AKT*. Mutations in *KRAS* (exons 1 and 2), *BRAF* (exons 11 and 15), *PIK3CA* (exons 9 and 20) and *AKT* (exon 3) were examined using a previously published DNA endonuclease (Surveyor™) based method (Jänne *et al*, 2006). Exon-specific PCR amplification was carried out for each of the indicated genes, the resulting DNA products were subjected to Surveyor™ digestion and analysed using the Transgenomic WAVE HS system as previously described (Jänne *et al*, 2006). All mutations were independently confirmed. PCR primers and conditions are available upon request.

### Statistical analyses

Statistical analyses were conducted using biomarker results obtained on primary tumours, except in 20 cases where only tissue from metastasis was available. The primary end point of the study was the identification of biomarkers potentially associated with progression of disease under cetuximab therapy. Analysis of ROC (receiver operating characteristic) curve was carried out with the aim of determining a cutoff point for *MET* and *IGF1R* expression or gene copy numbers as a continuous variable (Cai and Moskowitz, 2004). Sensitivity and specificity were expressed in terms of percentage and the value for which sensitivity and sensibility were the highest has been chosen as the best cutoff point. Secondary end points were association with TTP and overall survival (OS). TTP was calculated from the time of first cetuximab infusion to time of disease progression or last disease assessment. OS was calculated from the time of first cetuximab infusion to patient death or last contact.

Differences in response rate were compared by Fisher's exact test or *χ*^2^ test. TTP, OS and the 95% confidence intervals were evaluated by survival analysis using Kaplan–Meier method (Kaplan and Meier, 1985). TTP and OS for the groups with negative and positive biomarker were compared using the log-rank test. Statistical significance was set at <0.05 for each analysis. All statistical analyses were performed using SPSS version 11.5.1 (SPSS Italia srl, Bologna, Italy).

## Results

### MET analysis

*MET* FISH analysis was successfully performed in 76 patients (Table 1). Receiver operating characteristic curve analysis was performed to identify the *MET* copy number cutoff that better discriminates a resistant population but no association was found with drug sensitivity. *MET* increased gene copy numbers (*MET* FISH+) were then defined as mean ⩾5 per cell, which was observed in seven patients (9.2%), including two (2.6%) with gene amplification and five (6.6%) with high polysomy. Interestingly, all *MET* FISH+ cases were also *EGFR* FISH+, and such association was statistically significant (*P*=0.006). Nevertheless, *MET* FISH+ status was not associated with resistance to cetuximab therapy, and no difference in progressive disease rate was observed between *EGFR* FISH+/*MET* FISH+ and *EGFR* FISH+/*MET* FISH− (42.8 *vs* 37.5%, *P*=0.7).

### IGF1R analyses

**Figure 1:**
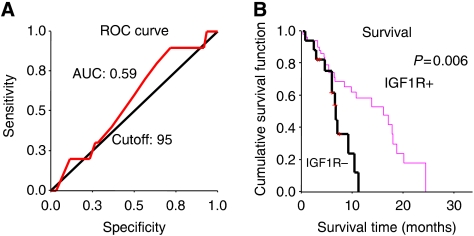
(**A**) ROC analysis identified an IGF1R value of 95 associated with a sensitivity of 90% (CI: 71.4–100) and a specificity of 28.3% (CI: 16.9–39.7). (**B**) Patients IGF1R positive (score⩾95) had a median survival of 16.1 months *vs* 6.7 months in IGF1R negative patients (*P*=0.006).

*IGF1R* was successfully analysed by FISH in 77 cases and by IHC in 70 cases. In the gene copy number evaluation, ROC analysis did not identify a cutoff discriminating sensitive *vs* resistant patient populations and no mCRC had mean >5 copies per cell to be called *IGF1R* FISH+. The mean *IGF1R* copy number ranged from 1.43 to 4.88 among the investigated mCRC. *IGF1R* gene copy number was not associated with IGF1R IHC expression (Pearson correlation coefficient 0.034, *P*=0.7). As shown in Figure 1A, the ROC analysis found an IGF1R IHC value of 95 associated with a sensitivity of 90% (CI: 71.4–100) and a specificity of 28.3% (CI: 16.9–39.7). Using this cutoff, 18 patients (25.7%) resulted negative and 52 (74.3%) positive (Table 2). Although response rate and TTP were not significantly different in the two groups, IGF1R IHC+ patients had a significantly longer survival than IGF1R IHC− (16.1 *vs* 6.7 months, *P*=0.006), as shown in Figure 1B. No difference in progressive disease rate was observed in *EGFR* FISH+/IGF1R IHC+ (N=26) *vs EGFR* FISH+/IGF1R IHC− (N=9; 42.3 *vs* 44.4%, *P*=0.9).

### Mutation analyses

*KRAS* analysis was successfully performed in 80 cases, and 42 patients (52.5%). harboured a mutation, with G13D as the most frequent mutation type (eight cases, 10.0%). Such mutations were not associated with clinical or biological characteristics and occurred in 22 *EGFR* FISH+ patients (53.7%). As illustrated in Table 3, response rate significantly favoured patients with *KRAS* wildtype (*P*=0.048), as well as TTP and OS, although these differences were not statistically significant (*P*=0.2 and 0.3, respectively).

*BRAF* was analysed in 79 cases, and only four patients (5.1%) harboured a mutation (V599E). *BRAF* and *KRAS* mutations were mutually exclusive. Owing to the low number of patients carrying a *BRAF* mutation the statistical power was not enough to detect difference in outcome. Nevertheless, none of the four *BRAF*-mutated patients responded to cetuximab therapy, and TTP and OS resulted shorter than in *BRAF* wild-type individuals (TTP: 1.2 *vs* 5.4 months, *P*=0.09; OS: 5.4 *vs* 9.8 months, *P*=0.3).

A total of 79 patients were analysed for *PI3KCA* mutations. The gene was mutated in 14 (17.7%) cases, with H1047 as the most frequent mutation type (seven cases, 8.8%). No difference in response rate, TTP or OS was observed in patients harbouring a *PI3KCA* mutation *vs* wild type individuals. No *AKT* mutation was identified among the 82 patients evaluated for this gene.

### Outcome according to mutation and *EGFR* FISH status

To determine the relevance of *KRAS* or *BRAF* mutation in the presence or absence of the drug target (*EGFR*), we analysed the outcome of patients according to *EGFR* FISH status (Table 4). *KRAS* mutations did not impact the outcome of *EGFR* FISH− patients. No difference in response rate (5.3 *vs* 0%, *P*=0.4), TTP (3.2 *vs* 3.5 months, *P*=1) and OS (10.8 *vs* 7.8 months, *P*=0.5) was observed in *EGFR* FISH−/*KRAS* wild type *vs EGFR* FISH−/*KRAS* mutated. Conversely, *EGFR* FISH+/*KRAS* mutated had a significantly lower response rate (18.2 *vs* 47.7%, *P*=0.04) and shorter TTP (5.3 *vs* 7.4 months, *P*=0.2) than *EGFR* FISH+/*KRAS* wild-type patients. Interestingly, four *EGFR* FISH+ patients with *KRAS* mutations (G12V in one case, G12D in another one, G13D in the remaining two cases) responded to cetuximab therapy. These patients had a mean EGFR gene copy number ranging from 3.03 to 4.03.

Similar results were obtained when the analysis was extended to patients with *KRAS* or *BRAF* mutation. No difference in cetuximab sensitivity were observed in *EGFR* FISH− irrespective of *KRAS* and *BRAF* status, while significantly lower response rate (16.7 *vs* 50.0%, *P*=0.037), shorter TTP (5.4 *vs* 7.8 months, *P*=0.12) and shorter OS (9.5 *vs* 17.9 months, *P*=0.13) was observed in *EGFR* FISH+/*KRAS* or *BRAF* mutated *vs EGFR* FISH+/*KRAS* or *BRAF* wild type.

## Discussion

In the present study, the first conducted in a mCRC population with known *EGFR* status, we showed that *KRAS* mutation is significantly associated with cetuximab failure in *EGFR* FISH+ patients, even if it does not preclude response. Although preclinical data supported the negative impact of *MET*, *IGF1R*, and *PI3KCA* on cetuximab activity, in our study none of them demonstrated a clinical relevance as predictors for primary resistance, irrespective of the *EGFR* FISH result.

Previous studies demonstrated that *KRAS* mutations represent the most relevant mechanism responsible for anti-*EGFR* strategy failure (Lièvre *et al*, 2006, 2008; Di Fiore *et al*, 2007; Khambata-Ford *et al*, 2007; Amado *et al*, 2008; De Roock *et al*, 2008). Nevertheless, in CRC no data existed whether *KRAS* mutations drove cetuximab resistance in a patient population potentially sensitive to anti-*EGFR* agents, neither whether such events occurred in *EGFR* FISH+ individuals. In the present study, we observed that the incidence of *KRAS* mutation was similar in *EGFR* FISH+ and FISH− subgroups, indicating that these phenomena are not mutually exclusive. It is noteworthy that no difference in outcome was observed in the group of patients *EGFR* FISH− irrespective of *KRAS* status, while the difference in response was significant only in *EGFR* FISH+. These results suggest that the drug maybe ineffective in absence of the target, while *KRAS* activation is able to drive drug resistance even if *EGFR* is present. Interestingly, in our population of *KRAS* mutated patients, four individuals responded to the therapy, and all were *EGFR* FISH positive. This finding raises the possibility that, in presence of an overexpressed target, occasionally tumour shrinkage could occur also in individuals harbouring the mutation.

*BRAF* mutations were identified in 66% of melanomas, and in smaller percentage in other human cancers including colorectal (Davies *et al*, 2002). Our study confirmed that such mutation is not frequent in CRC and could represent an additional mechanism responsible for cetuximab failure. None of the patients carrying *BRAF* mutation responded to the therapy, with a trend for shorter TTP and survival, differences not significant probably because of the small number of patients.

A recent study showed that colorectal cancer cell lines with activating *PI3KCA* mutations or with loss of PTEN expression were more resistant to cetuximab therapy than *PI3KCA* wild type/PTEN expressing cell lines (Jhawer *et al*, 2008). Importantly, simultaneous mutations of *KRAS* and *PI3KCA* conferred maximal resistance to cetuximab. In our study, combination of multiple mutation tests (*KRAS or BRAF*±*PI3KCA*) did not provide additional information over a single mutation test (data not shown).

Previous studies have investigated *MET* in CRC with semiquantitative techniques such as immunoblotting or IHC (Di Renzo *et al*, 1995; Hiscox *et al*, 1997; Fujita and Sugano, 1997; Fukuura *et al*, 1998). Our study represents the first report in CRC assessing *MET* at the genomic level using FISH. We report here that *MET* amplification is a rare event in CRC, occurring in about 2% of cases. Only two patients had *MET* amplification and both responded to cetuximab therapy. Although the number of patients was too low for any conclusion, the level of *MET* gene gain observed in our study population was lower than reported in a previous study conducted on cell lines and patients with acquired resistance to anti EGFR agents (Engelman *et al*, 2007). Although obtained in tissues collected before starting cetuximab therapy, these findings suggest that only high level of *MET* gene gain could be responsible for resistance, levels probably occurring only under therapeutic pressure and rarely present in a general population of patients unexposed to anti-EGFR agents.

IGF1R is overexpressed in 50–90% of CRC (Weber *et al*, 2002; Koda *et al*, 2004), and preclinical studies suggested that this target could be responsible for resistance to anti-*EGFR* strategies (Chakravarti *et al*, 2002). In our study, IGF1R expression was not associated with cetuximab resistance, probably because the IGF1R pathway did not affect the antiproliferative activity of cetuximab, as recently observed in a lung cancer model (Morgillo *et al*, 2007). In the study conducted by Morgillo *et al* (2007) only the treatment with gefitinib, an *EGFR*-TKI, but not cetuximab, induced *EGFR*-IGF1R heterodimerisation and activation of IGF1R and its downstream signalling mediators, resulting in increased survivin expression in NSCLC cell lines with high levels of IGF1R expression. Interestingly, IGF1R-expressing patients had longer survival than IGF1R negative. A similar pattern has been observed in other malignancies such as non-small cell lung cancer or soft tissue sarcomas (Ahlén *et al*, 2005; Cappuzzo *et al*, 2006), whereas the opposite situation was found in other malignancies such as uveal melanoma and breast cancer (Turner *et al*, 1997; All-Ericsson *et al*, 2002). The significant association with survival is of particular relevance and merits further investigations, considering the number of strategies interfering with the IGF1R pathway under evaluation in solid cancers, including CRC. In our study, no patient had *IGF1R* gene amplification and the level of *IGF1R* gene gain was very low in the whole analysed population, suggesting that such event is not involved in primary resistance. Moreover, no association was found between *IGF1R* gene and protein expression, suggesting post-transcriptional events could also interfere with the gene function.

To conclude, the present study showed that presence of *KRAS* mutations represents the strongest predictor for cetuximab failure in *EGFR* FISH-positive CRC patients. The rarity of *MET* and *IGF1R* gene amplification suggest that such biological events play a limited role in primary resistance to anti-*EGFR* agents. The impact of *BRAF* mutation on cetuximab resistance as well as the potential prognostic implications of IGF1R expression requires further investigation.

## Figures and Tables

**Table 1:** Association of MET gene copy number with outcome to cetuximab therapy and patient biomarker status

	**Total (*N*/%)**	**MET FISH+ (*N*/%)**	**MET FISH− (*N*/%)**	***P*-value**
Total evaluated	76	7	69	
Progressive disease	38/50.0	3/42.8	35/50.7	0.7
Complete+partial+stable disease	38/50.0	4/57.2	34/49.3	
Evaluated for EGFR Copy number	76	7	69	
*EGFR* FISH+	39/51.3	7/100	32/46.3	0.006
*EGFR* FISH−	37/48.7	0	37/53.7	
Evaluated for KRAS mutation	75	6	69	
*KRAS* mutated	40/53.3	2/28.5	38/55.1	0.3
*KRAS* wild type	35/46.7	4/57.2	31/44.9	
Evaluated for IGF1R expression	63	6	57	
IGF1R IHC+	48/76.1	4/66.6	44/77.1	0.5
IGF1R IHC−	15/23.9	2/33.4	13/22.9	
				

A mean ⩾5 *MET* gene copy number qualified the sample as *MET* FISH positive. A mean ⩾2.92 *EGFR* gene copy number qualified the sample as *EGFR* FISH positive. Among the 76 patients evaluated for *MET*, all were evaluated for *EGFR* FISH, 75 were evaluated for presence of *KRAS* mutations, and 63 for IGF1R expression.

**Table 2:** Association of IGF1R expression with patient biomarker status and outcome to cetuximab therapy

	***N*/%**	**IGF1R IHC+ (*N*/%)**	**IGF1R IHC− (*N*/%)**	***P*-value**
Total evaluated	70	52	18	
Progressive disease	35/50.0	24/46.2	11/61.1	0.27
Complete+partial+stable disease	35/50.0	28/53.8	7/38.9	
Evaluated for EGFR copy number	70	52	18	
*EGFR* FISH+	35/50.0	26/50.0	9/50.0	1.0
*EGFR* FISH−	35/50.0	26/50.0	9/50.0	
Evaluated for KRAS mutation	67	51	16	
*KRAS* mutated	33/47.1	27/51.9	6/33.3	0.28
*KRAS* wild type	34/48.5	24/46.1	10/55.5	
Evaluated for MET copy number	63	48	15	
MET FISH+	6/8.5	4/7.6	2/11.1	0.5
MET FISH−	57/81.4	44/84.6	13/72.2	
				

Using an ROC analysis, a score ⩾95 qualified the tumour as IGF1R positive. A mean ⩾5 *MET* gene copy number qualified the sample as *MET* FISH positive. A mean ⩾2.92 *EGFR* gene copy number qualified the sample as *EGFR* FISH positive. Among the 70 patients evaluated for IGF1R, all were evaluated for *EGFR* FISH, 67 for *KRAS* mutations, and 63 for *MET* gene copy number.

**Table 3:** *KRAS*, *BRAF*, and *PI3KCA* mutations and outcome to cetuximab therapy

**Genes**	**Total**	**Response rate (*N*/%)**	**Time to progression (months)**	**Survival (months)**
*KRAS* evaluated	80			
*KRAS* mutated	42	4/9.5	4.4	9.5
*KRAS* wild type	38	10/26.3	5.4	10.8
*P-value*		*0.048*	*0.2*	*0.3*
*BRAF* evaluated	79			
*BRAF* mutated	4	0	1.2	5.4
*BRAF* wild type	75	13/17.3	4.6	9.8
*P-value*		*0.3*	*0.09*	*0.3*
PI3KCA evaluated	79			
PI3KCA mutated	14	4/28.6	6.3	9.5
PI3KCA wild type	65	9/13.8	4.6	9.8
*P-value*		*0.2*	*0.2*	*0.7*

**Table 4:** Outcome of patients with *KRAS* or *BRAF* mutations stratified according to *EGFR* FISH status

	**Total**	**Response rate (*N*/%)**	**Time to progression (months)**	**Survival (months)**
*EGFR* FISH+/*KRAS*+	22	4/18.2	5.3	13.8
*EGFR* FISH+/*KRAS*−	19	9/47.4	7.4	9.8
*P-value*		*0.04*	*0.2*	*0.3*
*EGFR* FISH−/*KRAS*+	20	0	3.5	7.8
*EGFR* FISH−/*KRAS*−	19	1/5.3	3.2	10.8
*P-value*		*0.4*	*1.0*	*0.5*
*EGFR* FISH+/*KRAS+* or *BRAF+*	24	4/16.7	5.4	9.5
*EGFR* FISH+/*KRAS*− and *BRAF−*	16	8/50.0	7.8	17.9
*P-value*		*0.03*	*0.1*	*0.1*
*EGFR* FISH−/*KRAS+* or *BRAF+*	22	0	3.4	7.8
*EGFR* FISH−/*KRAS*− and *BRAF−*	17	1/5.9	3.8	10.8
*P-value*		*0.4*	*0.5*	*0.5*

KRAS+: Presence of mutation; KRAS−: No mutation found.

BRAF+: Presence of mutation; BRAF−: No mutation found.
